# MicroRNA-4287 is a novel tumor suppressor microRNA controlling epithelial-to mesenchymal transition in prostate cancer

**DOI:** 10.18632/oncotarget.27849

**Published:** 2020-12-22

**Authors:** Divya Bhagirath, Thao Ly Yang, Theresa Akoto, Nikhil Patel, Laura Z. Tabatabai, Sharanjot Saini

**Affiliations:** ^1^Veterans Affairs Medical Center, University of California, San Francisco, CA, USA; ^2^Department of Biochemistry and Molecular Biology, Augusta University, Augusta, GA, USA; ^3^Department of Cellular Biology and Anatomy, Augusta University, Augusta, GA, USA; ^4^Department of Pathology, Augusta University, Augusta, GA, USA

**Keywords:** miR-4287, prostate cancer, chromosome 8p, EMT, SLUG

## Abstract

Prostate cancer (PCa) is a significant cause of male morbidity in the United States. Despite recent advances in diagnosis and therapeutic interventions, significant fraction of cases still progress to an advanced stage. Various genetic/epigenetic elements that facilitate this progression are not yet completely known and the mechanism that favors advanced disease is an area of investigation. A characteristic feature associated with progressive disease is deletion of chromosome 8p (chr8p) region, that harbors tumor-suppressor *NKX3.1*. Previous studies from our group has shown that there are cluster of microRNAs (miRNAs) located within this region whose loss favors advanced, metastatic disease. miR-4287 is a novel miRNA located within this region that has not been studied before. In the present study, we analyzed the role of miR-4287 in PCa using clinical tissues and cell lines. We observed that miR-4287 is significantly downregulated in patient-derived tumor tissues. Receiver operating curve (ROC) analysis showed that miR-4287 distinguishes prostate cancer from normal with a specificity of 88.24% and with an Area under the curve (AUC) of 0.66. Further, we found that miR-4287 levels correlate inversely with patients’ serum prostate-specific antigen levels. Ectopic over-expression of miR-4287 in PCa cell lines showed that miR-4287 plays a tumor suppressor role. miR-4287 led to an increase in G2/M phase of cell cycle in PCa cell lines. Further, ectopic miR-4287 inhibited PCa epithelial-to-mesenchymal transition (EMT) by directly repressing SLUG and stem cell marker CD44. Since miR-4287 specifically targets metastasis pathway mediators, miR-4287 has potential diagnostic and therapeutic significance in preventing advanced, metastatic disease.

## INTRODUCTION

Prostate cancer (PCa) is the second leading cause of cancer related deaths among men in the United States. In 2020, an estimated 191,930 PCa cases are expected to be reported in the United States and among them, 33,330 are projected to die from the disease [1, 2]. Tissue biopsy is the standard measure for diagnosis of prostate cancer. Based on the Gleason score of the microscopically analyzed biopsy, clinicians stratify the disease as low, intermediate or high risk [2]. In addition to these, advanced imaging and biomarker analysis is another accepted measure to understand the disease risk. Prostate Specific Antigen (PSA), a glycoprotein that is synthesized and released by normal and tumor cells, is often used for early detection and diagnosis of prostate cancer. Its relative abundance is significantly increased in serum of individuals with PCa and therefore serve as an early indicator of disease. However, PSA is not a very specific PCa biomarker and is found to be elevated in patients with benign prostatic hyperplasia (BPH), that often leads to over-diagnosis and false positive results [3–5]. This has therefore led the for more specific biomarkers that can not only diagnose the disease but can also successfully predict disease outcome and stratify the disease severity [5, 6]. Towards this, significant advances have been made to better understand prostate cancer biology, including identification of genomic rearrangements and alterations, chromosomal translocations and deletions that are associated with prostate cancer [7–9]. These genetic anomalies are now being identified as a very specific indicator of disease severity and can predict the clinical course of the disease with a much higher accuracy [10].

Non-coding RNAs as microRNA (miRNAs) are emerging as another important molecular mediators of PCa biology as well as biomarkers for early disease diagnosis and risk stratification [6]. Chromosomal deletions/amplifications are often found in aggressive PCa [11, 12]. Loss of chromosome 8p21 region has been reported in advanced PCa [12]. This region, in addition to harboring a tumor suppressor gene *NKX3-1*, [13] contains a series of miRNAs that are eventually lost upon disease progression as a result of chromosomal deletion. Previous research from our laboratory has shown an important tumor suppressor role of these miRNAs including miR-3622a, miR-3622b, miR-383 and miR4288 whereby these miRNAs are down-regulated in prostate tumors, mediate an anti-proliferative effect on tumor cells and are involved in inhibiting the metastasis and progression of the disease [14–17]. miR-383 was found to regulate the cancer stem cell properties of PCa cells via regulation of CD44, that in turn regulates their metastatic abilities [14]. miR-3622a inhibits PCa invasiveness via regulating EMT mediators such as ZEB1 and SNAI2 [14, 15], while miR-3622b regulates proliferation and invasiveness by regulating Epidermal Growth Factor Receptor (EGFR) [16]. Most recently, we showed that miR-4288 regulates invasiveness via targeting MMP16 and ROCK1 in tumor cells and it is specifically altered in Caucasians as compared to African Americans suggesting a race-specific alteration [17].

In the present study, we studied the function of a novel miRNA, miR-4287, another miRNA that falls on chromosome 8p 21.1 (GRCh38.p13) within intron of gene scavenger receptor class A member 5 (SCARA5), in prostate cancer cell lines. To our knowledge, there are no reports of miRNA-4287 in PCa till date and its role in prostate cancer is largely unknown. We observed a similar tumor suppressor role of miR-4287 in prostate cancer as it was found to be downregulated in PCa clinical samples. Overexpression of miR-4287 in PCa cell lines led to an increase in G2/M phase of cell cycle. Further, we found that miR-4287 targets mediators of EMT pathway and CD44, thereby suggesting an anti-invasive and anti-metastatic role of this miRNA in PCa pathogenesis. Taken together, our findings suggest that miR-4287, along with miR-3622a/b, miR-383, miR-320a [14–17], constitute a series of tumor suppressor miRNAs located on frequently deleted chr8p region. Our findings suggest that loss of these miRNAs lead to aggressive, metastatic PCa. These findings support the mechanistic role of chr8p region in tumor progression. Chr8p region has traditionally been associated with PCa initiation though a significantly higher deletion frequency has been reported in advanced PCa [18, 19] reference suggesting its role in PCa progression.

## RESULTS

### miR-4287 is downregulated in prostate cancer

**Figure 1:**
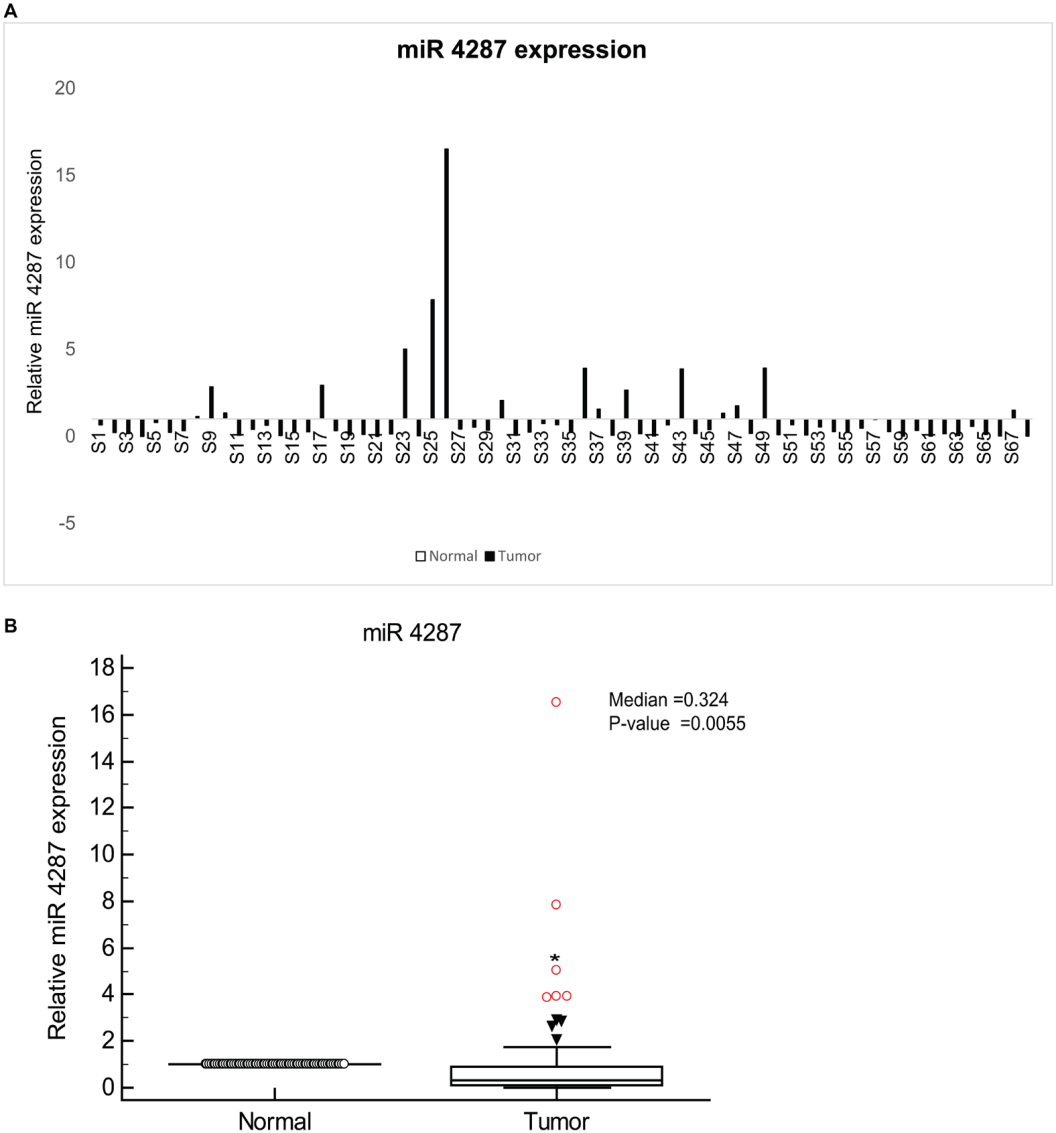
miR-4287 expression is widely attenuated in prostate cancer. (**A**) Relative miR-4287 expression in microdissected PCa tissues (*n* = 68) and patient matched normal adjacent tissues as assessed by real time PCR. Data was normalized to RNU48 controls. (**B**) Median miR-4287 expression in normal and tumor tissues in (A).

We first assessed the expression of miR-4287 in tumor tissues derived from 68 prostate cancer patients. Clinicopathological characteristics for the patients are given in Supplementary Table 1. Using adjacent normal tissue as the corresponding control for each tumor sample, we observed that miR-4287 expression was downregulated in almost 74% of the analyzed tumors (Figure 1A). The median expression for miR-4287 (0.324) in tumors was significantly lower than the corresponding normals (*P* = 0.0055) (Figure 1B).

### miR-4287 expression negatively correlates with serum PSA levels

**Figure 2:**
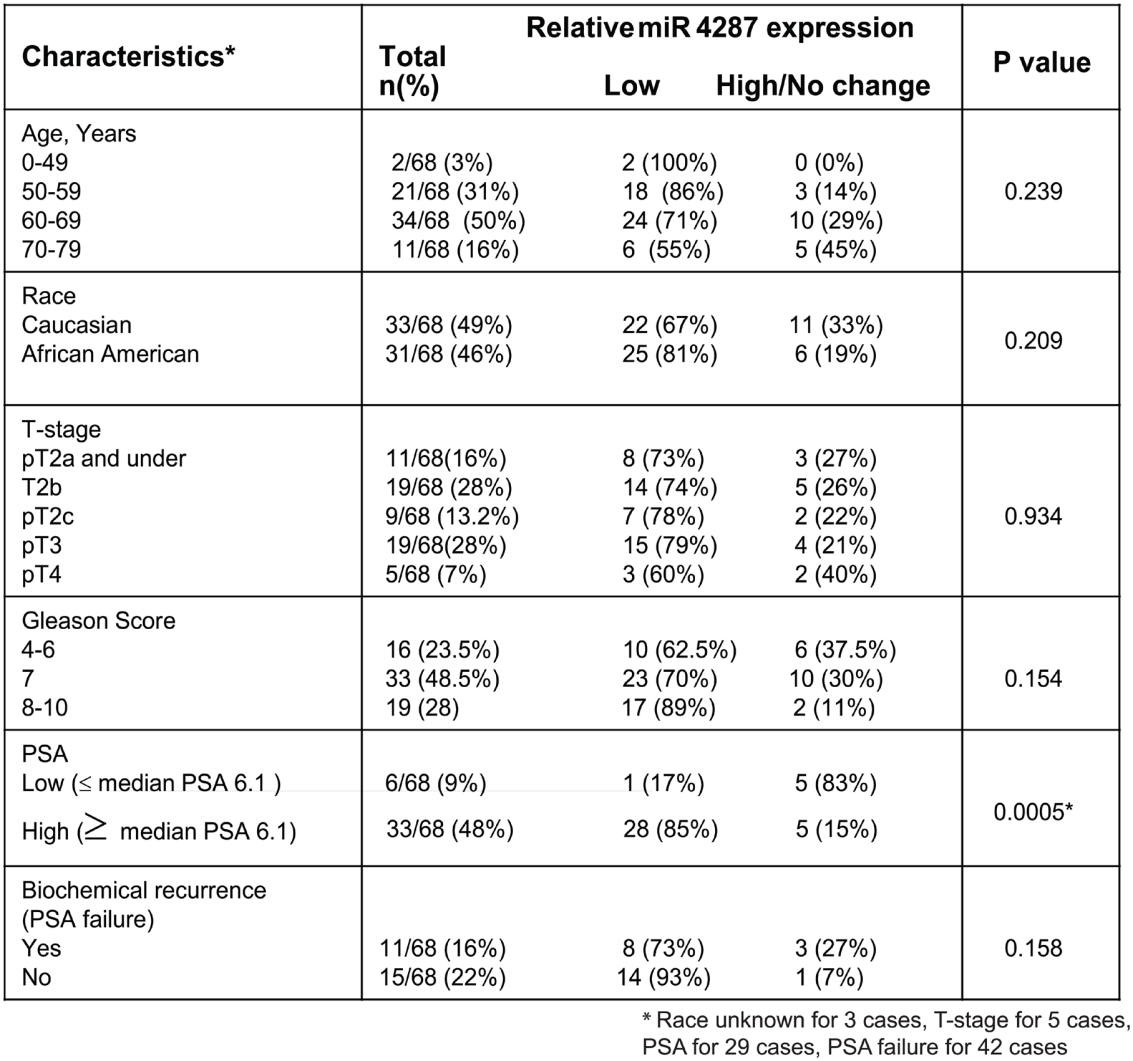
Correlation of miR-4287 expression with clinicopathological parameters of prostate cancer. miR-4287 expression in PCa patients correlated with age, race, pathological stage (T), Gleason score, PSA and biochemical recurrence. *P*-values were calculated by Chi square test. ^*^ *P* < 0.05.

To understand the relevance of miR-4287 with severity of disease and other clinical parameters, we assessed the correlation of miR-4287 expression with tumor stage, Gleason score, age, race, biochemical recurrence and PSA levels (Figure 2). Patients in lower age group had higher percentage of samples with low miR-4287 expression than those with higher age. Similarly, a trend was observed with increasing Gleason score with percentage of patients with low miR-4287 expression increasing from 62.5% in Gleason 4–6 to 70% and 89% in Gleason 7 and 8–10, respectively. Although there was some correlation between age, Gleason score and also race and biochemical recurrence and miR-4287 expression, these correlations failed to reach statistical significance based on our analysis (*P*-values = 0.154, 0.239, 0.209 and 0.158, respectively). Interestingly, we observed that miR-4287 expression significantly correlated with serum PSA levels (*P* = 0.0005). 85% of PCa patients with serum PSA levels higher than the median PSA value (6.1) for the cohort had a lower miR-4287 expression (Figure 2) as compared to 17% amongst patients with lower than median PSA expression.

### miR-4287 as a diagnostic biomarker for prostate cancer

**Figure 3:**
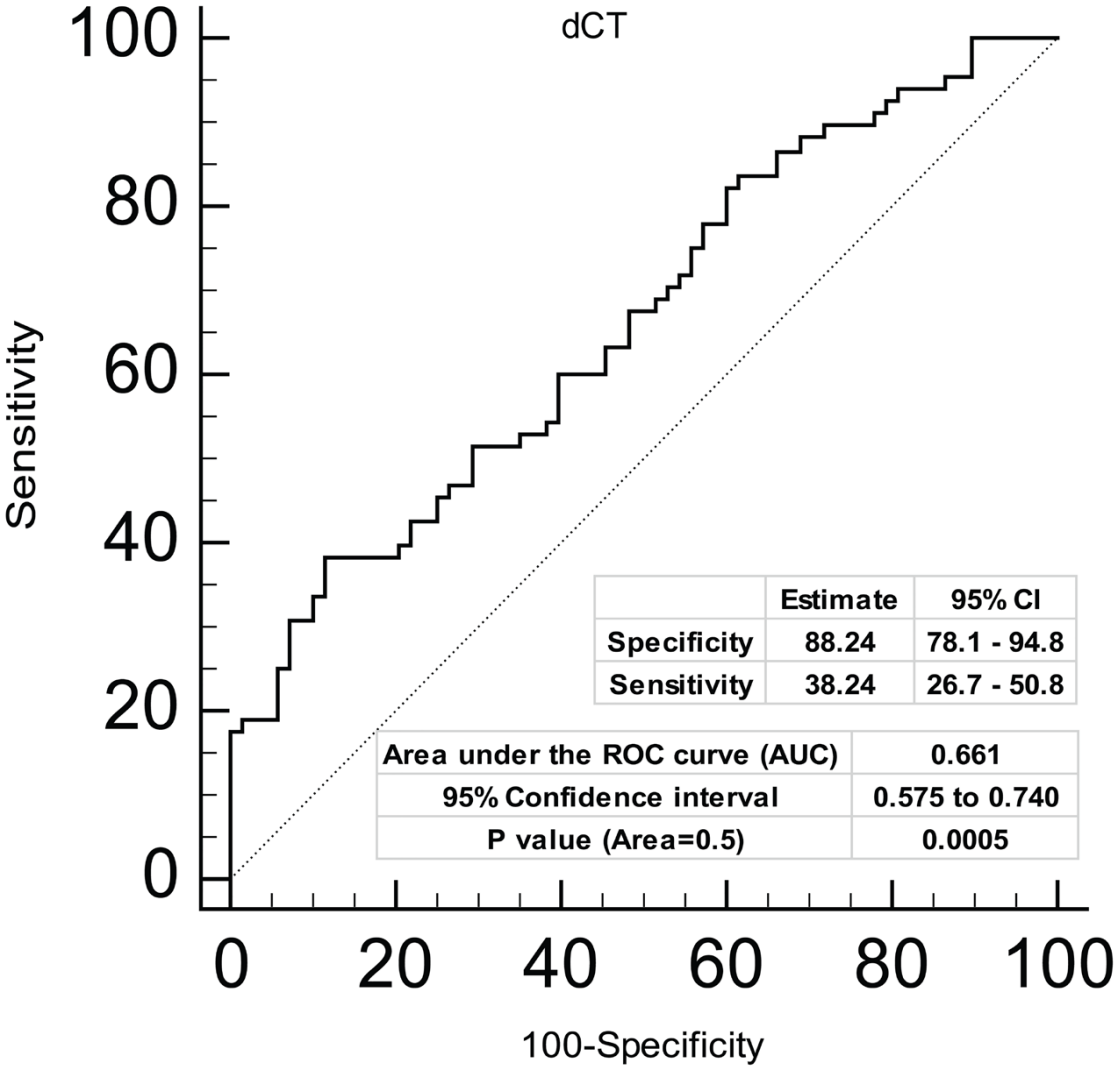
Diagnostic ability of miR-4287 expression in prostate cancer. ROC curve analysis showing the ability of miR-4287 expression to discriminate between tumor and adjacent normal tissues based on dCt values. ^*^ *P* < 0.05.

Further, in view of low miR-4287 expression observed in a high percentage of clinical tissues, we asked if miR-4287 expression can be used as a parameter for diagnosing prostate cancer. To test this, we performed receiver operating curve (ROC) analysis on the tested clinical samples (Figure 3). Our analyses based on dCt values on tumor and matched normal samples suggest that expression of miR-4287 can distinguish prostate cancer from the normal with 88.24% specificity and with an Area under the curve (AUC) of 0.66 (95% CI: 0.575 to 0.740), *P* = 0.0005 (Figure 3).

### miR-4287 over-expression causes increase in G2/M phase in prostate cancer cell lines

**Figure 4:**
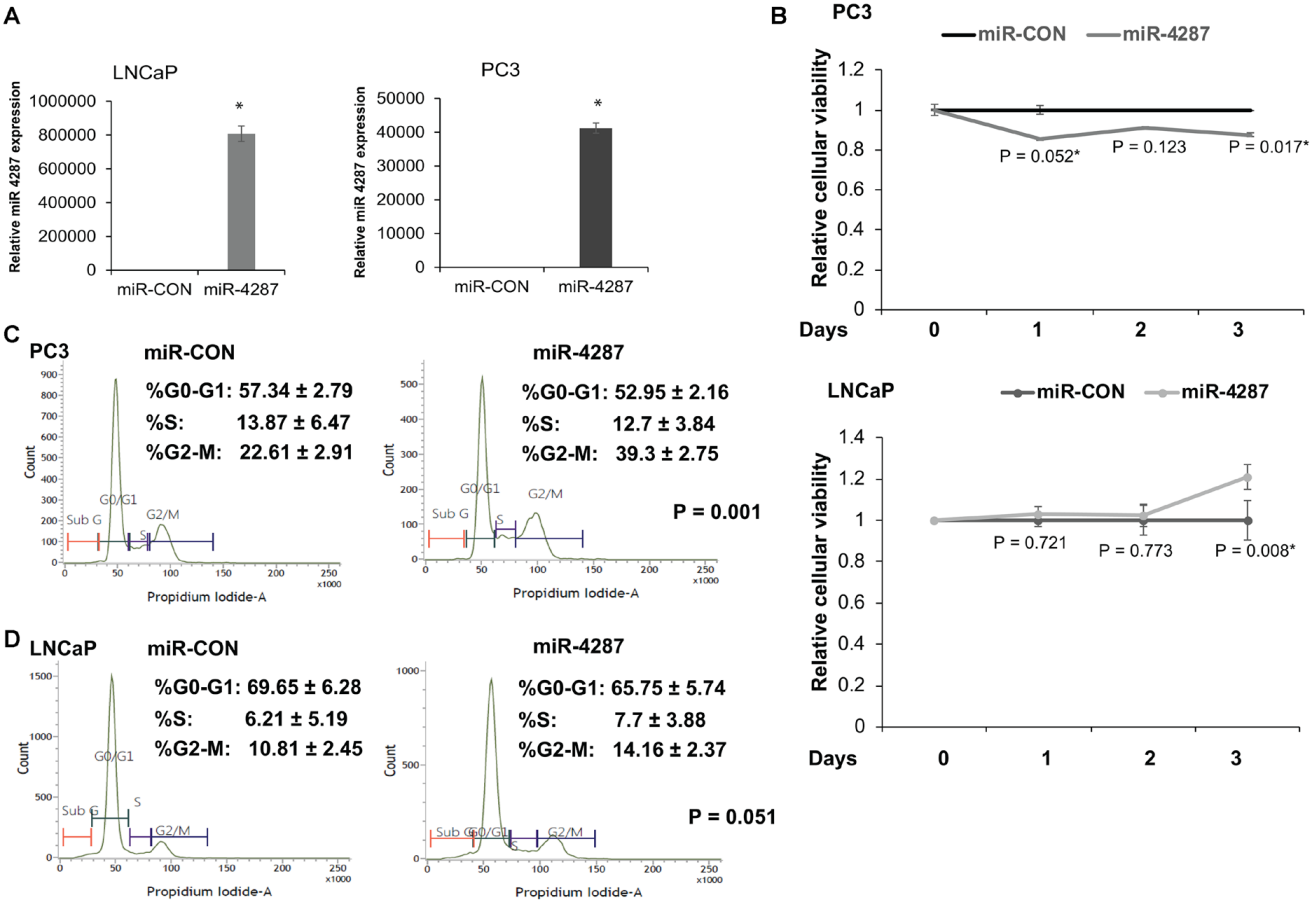
miR-4287 overexpression causes increase in G2/M phase in prostate cancer cell lines. (**A**) Left panel: Assessment of miR-4287 levels upon transient transfection of control miR (miR-CON)/ miR-4287 in PC3 cells by RT-PCR. Data were normalized to RNU48 levels. Right panel: Assessment of miR-4287 levels upon transient transfection of control miR (miR-CON)/ miR-4287 in LNCaP cells by RT-PCR. Data were normalized to RNU48 levels. (**B**) Cellular viabilities in PC3 (upper panel) and LNCaP cells (lower panel) upon control/miR-4287 transfections at indicated time points as measured by MTS assay. (**C**) Flow cytometry analyses of DAPI-stained miR-CON/miR-4287 transfected PC3 cells. (**D**) Flow cytometry analyses of DAPI-stained miR-CON/miR-4287 transfected LNCaP cells. ^*^ *P* < 0.05.

To understand the functional role of miR-4287 in prostate cancer, we ectopically overexpressed miR-4287 in PCa cell lines LNCaP and PC3 by transiently transfecting the cell lines with miR-4287 or miR-CON mimics. We performed real time PCR on PC3 or LNCaP cells transfected with miR-4287 or miR-CON (Figure 4A) and evaluated miR-4287 expression that confirmed its overexpression in miR-4287 transfected cells as compared to miR-CON transfected cells. Following transient transfections of miR-CON/miR-4287 mimics, cellular viability assays showed that in androgen independent PC3 cell line, miR-4287 overexpression causes significant decreases in cellular viabilities at Days 1 and 3 (Figure 4B, upper panel). However, in androgen dependent LNCaP cells, miR-4287 was found to exert a differential effect, with no significant changes at Days 1 and 2 and slight increase at Day 3 (Figure 4B, lower panel). Cell cycle analyses of cells in the two groups demonstrated a significant (*P* = 0.001 and *P* = 0.051 respectively) increase in the G2/M phase in both PC3 and LNCaP (Figure 4C and 4D) cell lines.

### miR-4287 over-expression regulates EMT in prostate cancer cell lines

**Figure 5:**
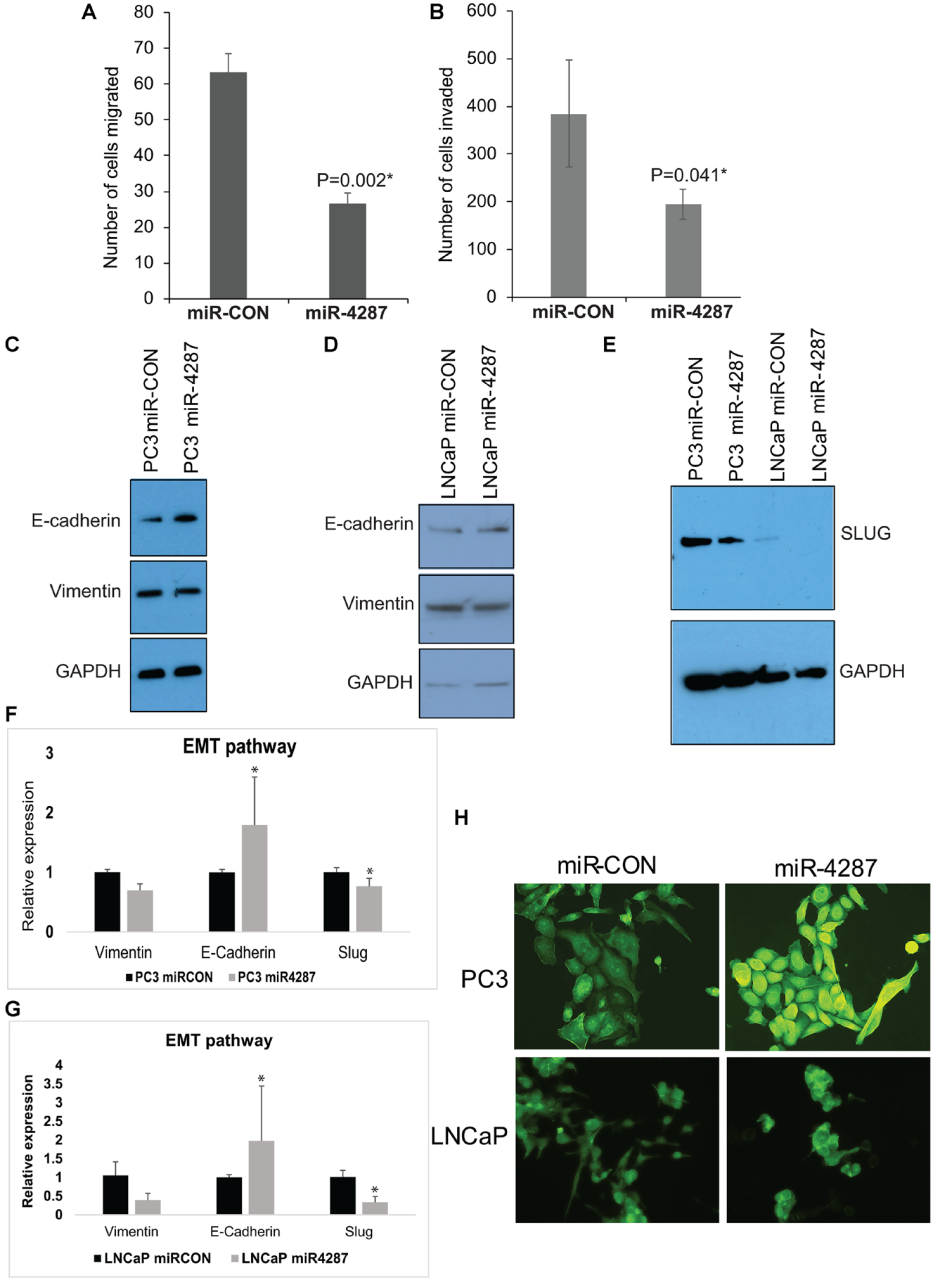
miR-4287 overexpression regulates EMT in prostate cancer cell lines. (**A** and **B**) Control miRNA/miR-4287 was transiently transfected in PC3 cells followed by. (A) *In vitro* transwell migration assay and, (B) *In vitro* transwell invasion assay. (**C**) Western blot analyses for E-cadherin and Vimentin in PC3 cells transfected with miR-CON/miR-4287. GAPDH was used as a loading control. (**D**) Western blot analyses for E-cadherin and Vimentin in LNCaP cells transfected with miR-CON/miR-4287. GAPDH was used as a loading control. (**E**) Western blot analyses for SLUG in PC3 and LNCaP cells transfected with miR-CON/miR-4287. GAPDH was used as a loading control. (**F**). Real time PCR based analyses of EMT markers *E-cadherin*/*CDH1*, *VIM* and *SLUG* in PC3 cells transfected with miR-CON/miR-4287. Data was normalized to *GAPDH* control. (**G**) Real time PCR based analyses of EMT markers *E-cadherin*/*CDH1*, *VIM* and *SLUG* in LNCaP cells transfected with miR-CON/miR-4287. Data was normalized to *GAPDH* control. (**H**) Morphological alterations in PC3 and LNCaP cells upon miR-CON/ miR-4287 transfections as assessed by FITC-labelled phalloidin staining. Magnification 40x. ^*^ *P* < 0.05.

**Figure 6:**
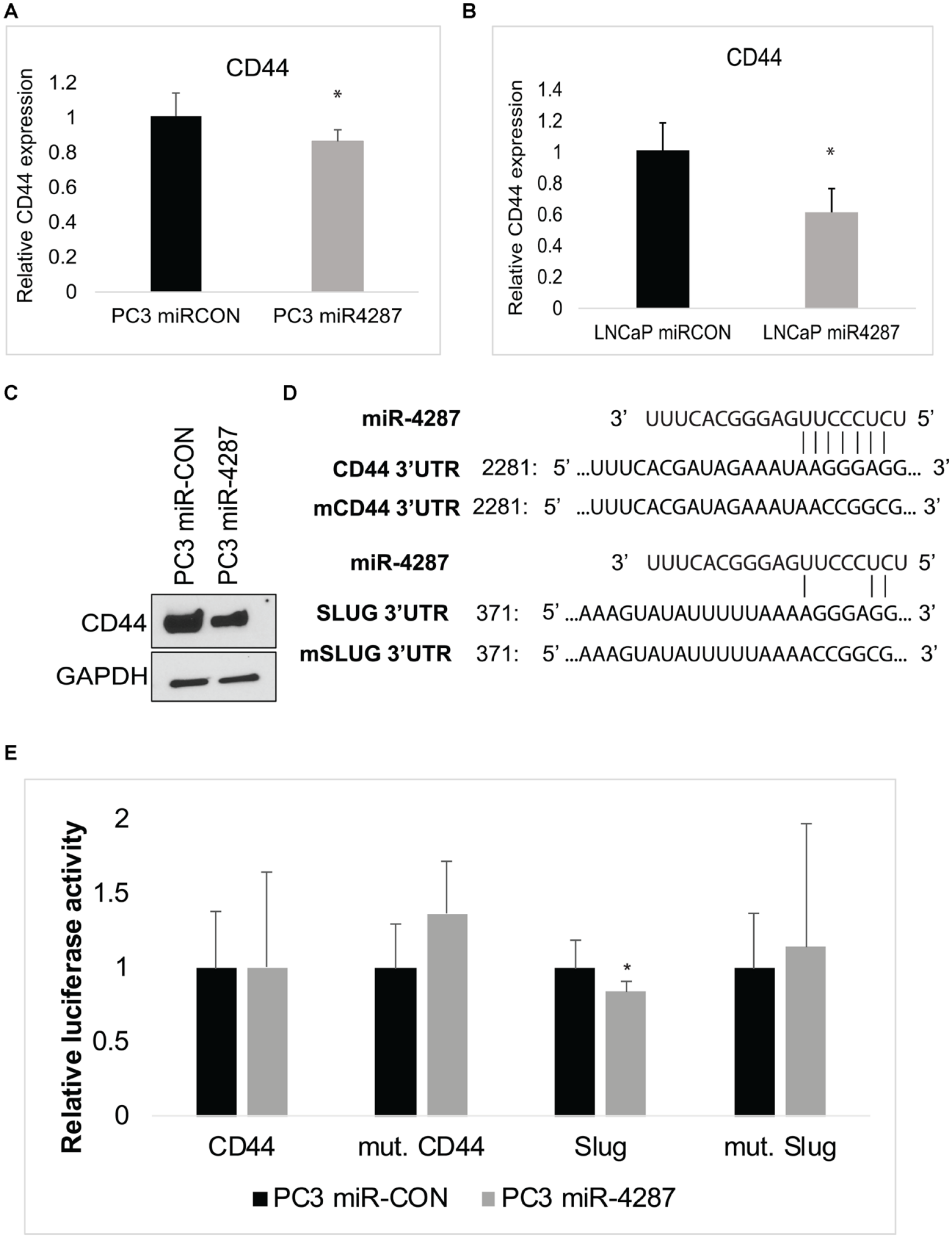
miR-4287 directly regulates stem cell marker CD44 and EMT mediator SLUG. (**A**) Real time PCR based analyses of *CD44* mRNA in PC3 cells transfected with miR-CON/miR-4287. Data was normalized to *GAPDH* control. (**B**) Real time PCR based analyses of *CD44* mRNA in LNCaP cells transfected with miR-CON/miR-4287. Data was normalized to *GAPDH* control. (**C**) Western blot analyses for CD44 in PC3 cells transfected with miR-CON/miR-4287. GAPDH was used as a loading control. (**D**) Schematic representation of 3′ UTR regions of CD44 and SLUG showing potential miR-4287 binding sites. Mutant 3′ UTR constructs employed in luciferase assay are shown below the wild-type (wt) 3′ UTR sequences. (**E**) Luciferase reporter assays with the indicated wt and mutant 3′ UTR constructs or control 3′ UTR construct co-transfected with miR-CON/miR-4287 transfected PC3 cells. Firefly luciferase values were normalized with Renilla luciferase and relative values were plotted. ^*^ *P* < 0.05.

We have previously observed an anti-invasive effect of Chr. 8p miRNAs- 3622a, 3622b, 383 and 4288 in prostate cancer cell lines. We tested the effect of miR-4287 overexpression on migratory and invasive properties of PCa cell line PC3 by *in vitro* transwell migration (Figure 5A) and invasion assays (Figure 5B). These assays showed that miR-4287 overexpression led to decreased migratory ability and invasiveness of PCa cells. Further, to test the effect of miR-4287 in regulating the EMT pathways, we analyzed protein expression of EMT markers – E-Cadherin and Vimentin in PC3 and LNCaP cells transfected with miR-4287 mimic. We observed an increase in E-Cadherin and decrease in Vimentin in PC3 cells (Figure 5C). In LNCaP cells, although we saw a decrease in Vimentin, increase in E-Cadherin was not consistently observed (Figure 5D). Upon examination of RNA expression, we observed an expected change in E-Cadherin and Vimentin in both PC3 and LNCaP cell lines (Figure 5F and 5G). Additionally, miR-4287 over-expression significantly downregulated EMT transcription factor Slug (Figure 5E–5G) which is also a direct target of miR-4287 in both the tested cell lines (Figure 6D). To further investigate the functional impact of miR-4287 on EMT pathway, we performed FITC-phalloidin staining on transfected cell lines. As expected, we found that cells overexpressing miR-4287 were more rounded and exhibited cortical staining pattern as compared to the controls suggesting that miR-4287 overexpression induces an epithelial form and reverses mesenchymal morphology of cancer cell lines (Figure 5H).

### miR-4287 targets cancer stem cell marker CD44 and EMT mediator SLUG in prostate cancer

To understand the biological function of miR-4287 in cancer cells, we further interrogated cellular targets for miR-4287 using TargetScan [20]. Upon examination, we identified CD44, as a direct target of miR-4287. To assess the impact of miR-4287 over-expression in prostate cancer cells, we evaluated the RNA expression of CD44 in PC3 and LNCaP cells transfected with miR-CON or miR-4287. We noticed a significant decrease in *CD44* mRNA expression in miR-4287 over-expressing cells (Figure 6A and 6B). Further, we assayed the protein expression of CD44 in miR-4287 transfected PC3 cells and observed a decrease in CD44 levels in miR-4287 over-expressing group (Figure 6C). We also assessed the changes in CD44 expression in LNCaP cells multiple times but the levels of CD44 in LNCaP cell line was very low and was not detectable upon western blot analysis. The 3′UTR of CD44 possesses a potential miR-4287 binding site (Figure 6D). Similarly, SLUG 3′ UTR has a miR-4287 binding site at position 371. To assess the direct binding of miR-4287 with its cellular targets *CD44* and *SLUG*, we performed a luciferase reporter assay with control/CD44 3′UTR constructs. PC3 cells transfected with miR-CON or miR-4287 were cotransfected with control/*CD44* and *SLUG* 3′UTR constructs and assayed for relative luciferase signal (Figure 6E). We observed a significant downregulation of *SLUG* expression upon miR-4287 overexpression that was reversed upon mutation of potential miR-4287 binding site, suggesting a direct binding of miR-4287 to *SLUG* 3′UTR to mediate its repression. However, CD44 may not be a direct miR-4287 target as co-transfection of miR-4287 with *CD44* 3′ UTR did not inhibit its luciferase reporter activity as compared to control 3′ UTR (Figure 6E). These data suggest that miR-4287 is another component of chr8p with a role in regulating EMT and stemness in PCa and that miR-4287 likely downregulates CD44 expression indirectly.

## DISCUSSION

Since their discovery in 1993 in *Caenorhabditis elegans* [21], miRNAs are now known to play pivotal roles in several normal cellular functions including differentiation, homeostasis, cell cycle, and apoptosis [22]. They are essential part of the cancer cell epigenetic regulatory machinery and confer these cells the ability to thrive and enable tumor growth and progression [23]. Previous discoveries from our laboratory have elucidated an essential role of these non-coding RNA molecules in PCa progression and metastasis [14–17, 23]. An important alteration associated with aggressive prostate cancer is loss of chromosome 8p region. Adding a significant step further in understanding prostate cancer progression, we identified that miRNAs that fall in the region of chromosome 8p are also lost with the progressing disease. miRNA-383, -3622a, -3622b and -4288 are significantly downregulated in PCa patients and these miRNAs function as tumor suppressors and play important role in preventing cancer cell growth, proliferation and metastasis [14–17]. In the present study, we analyzed the role of a novel miRNA- miR-4287, located on chromosome 8p deleted region, in prostate cancer pathogenesis and report for the first time that miR-4287 is dysregulated in prostate carcinomas.

Like other miRNAs that belong to this deleted chromosome region, we observed a significant decrease in miR-4287 expression in microdissected tissues from PCa patients at different stages of the disease. Although, we did not observe any significant correlation between loss of miR-4287 expression with the increasing tumor stage and pathological grade in prostate cancer, we noticed that increasing serum PSA levels correlated negatively with miR-4287 expression. PSA is a serine protease, that is under the regulatory control of Androgen receptor (AR) and is induced by AR under both androgen dependent and independent conditions [24]. AR is widely known to control the transcriptional landscape of prostate cancer cell [25]. An important component of this regulatory control includes regulation of miRNAs by AR either by indirectly controlling the epigenetic machinery or directly acting on the promoters of miRNA gene [26, 27]. Additionally, different miRNAs are also known to control AR, whereby miRNAs downregulate AR by acting on AR 3′UTR [27, 28]. The inverse relationship between PSA and miR-4287 indicates a possible regulatory control of miR-4287 via AR pathway or vice versa. We examined the interplay between androgens/AR and miR-4287 expression and found that upon induction of PC3 and LNCaP cells with AR agonist dihydrotestosterone (DHT), miR-4287 expression is downregulated (Supplementary Figure 1), pointing to its regulation via androgens/AR axis. Although, AR is not a direct target for miR-4287, it may through indirect mediators control the levels or activity of AR. Understanding this axis and how it may alter prostate cancer pathogenesis can be a further area of investigation.

Despite this possible regulation of miR-4287 by AR, we observed a similar downregulation of EMT pathway mediators and stem cell marker CD44 in both AR negative (PC3) and AR positive (LNCaP) cell line emphasizing its tumor-suppressor role in prostate cancer. Importantly, we demonstrate for the first time, that miR-4287 is a key negative regulator of prostate cancer stemness marker CD44 and EMT mediator SLUG. Since CD44 has been reported to play critical roles in cancer stemness and prostate cancer metastasis [29] and SLUG is an EMT mediator, we propose that frequent loss of miR-4287 promotes PCa EMT, stemness and metastasis via its regulation of CD44 and SLUG. SLUG, a repressor of E-cadherin, is also known to be regulated by several miRNAs including miR-3622a [15]. We previously reported that CD44 is regulated by miR-383 [14], a chr8p miRNA, and miR-708 [30] in prostate cancer. In addition, CD44 has been previously reported to be regulated by miR-34a [31], miR-373 and miR-520c [32] in prostate cancer. miR-34a was found to be a key negative regulator of CD44+ PCa cells and thereby, promote PCa development and metastasis [31]. We found decreased CD44 mRNA and protein levels upon miR-4287 overxpression as compared to corresponding controls although results from luciferase reporter assays showed that CD44 may not be a direct miR-4287 target. These data suggest that miR-4287 likely represses CD44 indirectly, via its effects on inhibiting EMT. It has been reported previously that EMT promotes CD44 expression and mesenchymal genes such as SLUG are positively correlated with CD44 expression while epithelial marker E-cadherin is involved in negative regulation of CD44 expression [33, 34].

In conclusion, in the present study we define a tumor-suppressor role of a novel miRNA- miR-4287- in prostate cancer via its regulation of prostate cancer EMT and stemness. This role of miR-4287 is in line with our earlier defined tumor suppressive role of other miRNAs located within this frequently deleted region on chromosome 8p [14–17, 23], implicating an important mechanistic role of chr8p in driving prostate cancer progression, metastasis and tumor recurrence. Given that these miRNAs play essential roles in PCa progression and are lost in advanced prostate cancer, it will be important to devise strategies to re-instate their expression in tumors via therapeutic interventions to successfully treat aggressive prostate cancer.

## MATERIALS AND METHODS

### Cell lines and cell culture

Prostate carcinoma cell lines LNCaP (RRID:CVCL_0395) and PC3 (RRID:CVCL_0035) were obtained from the American Type Culture Collection (ATCC) and cultured under recommended conditions. LNCaP and PC3 cell lines were maintained in RPMI 1640 media (Invitrogen) supplemented with 10% fetal bovine serum (FBS) (Atlanta biologicals) and 1% penicillin/streptomycin. Both the cell lines were maintained in an incubator with a humidified atmosphere of 95% air and 5% CO_2_ at 37°C. The experiments with cell lines were performed within 6 months of their procurement/resuscitation. Prostate cell lines were authenticated by DNA short-tandem repeat analysis. Cells were periodically tested for mycoplasma contamination by DAPI staining.

### Tissue samples

Formalin-fixed, paraffin-embedded (FFPE) PCa samples were obtained from the SFVAMC and Augusta University. Written informed consent was obtained from all patients and the study was approved by the UCSF and Augusta University Committees on Human Research. All slides were reviewed by a board-certified pathologist for the identification of PCa foci as well as adjacent normal glandular epithelium. Microdissection of tissues was performed as described earlier [35]. Briefly, 8 μm sections were placed on glass slides, deparaffinized by xylene, hydrated with gradient of alcohol, stained with hematoxylin, dehydrated, and microdissected under a light microscope [35].

### RNA extraction from FFPE tissues and cultured cells

RNA was extracted from microdissected FFPE tissues and cultured cells using a miRNeasy FFPE Kit (Qiagen) and an miRNeasy mini kit (Qiagen), respectively following the manufacturer’s instructions as described in [35].

### Quantitative real-time PCR

Mature miRNA miR-4287 (assay ID 479779_miR) was assayed using the TaqMan MicroRNA Assay, in accordance with the manufacturer’s instructions (Applied Biosystems). miRNA expression was normalized to RNU48 control (assay ID 001006) (Applied Biosystems). Relative changes in gene expression were calculated by comparative Ct method with the 7500 or StepOnePlus Fast Real Time PCR System (Applied Biosystems).

### miRNA transfections

Cells were plated in growth medium without antibiotics ~24 hrs before transfections. Transient transfections were carried out by miRNA precursors (Ambion) by using Lipofectamine 2000 (Invitrogen) according to the manufacturer’s protocol. Control miRNA (miR-CON; AM17110)/miR-4287 precursor (PM18771) was used for miRNA transfections followed by functional assays. All miRNA transfections were for 72 h.

### *In vitro* migration and invasion assays

Control inserts (for migration) or Matrigel inserts (for invasion) (BD Biosciences) were used for performing *in vitro* migration and invasion assays as per manufacturer’s protocol. Briefly, 48 hrs after transient transfections with miR-CON/miR-4287, cells were trypsinized, harvested and counted. Then 50,000 cells in a volume of 500 μl serum-free medium were each placed on Matrigel or control inserts and cells were allowed to migrate at 37°C for 24 hours. Following this, cells on the top of the inserts were removed with cotton swabs and cells that migrated/invaded though the polycarbonate/basement membrane were fixed, stained with Geimsa followed by counting of stained cells under light microscope. Five different fields were counted for each insert and average of cell count from these five fields were used to derive average cells migrated/invaded under each condition.

### Cellular viability assays

Cellular viabilities were determined at 24, 48, 72 hours post-transfection by employing the CellTiter 96 AQueousOne Solution Cell Proliferation Assay Kit (Promega), according to the manufacturer’s protocol.

### Cell cycle analyses

Fluorescence-activated cell-sorting (FACS) for analyzing cell cycle was done 72 hours post-transfection. Cells were harvested, washed with cold PBS, and resuspended in the nuclear stain DAPI (Beckman Coulter) for cell cycle analysis according to the manufacturer’s protocol. Stained cells were immediately analyzed by FACS (Cell Lab Quanta SC; Beckman Coulter, Inc).

### FITC-phalloidin staining

Cells were transfected with miR-CON/miR-4287 precursor. After 72 hrs, cells were fixed with 4% paraformaldehyde for 15 min and stained with Fluorescein isothiocyanate (FITC) -labelled phalloidin (Sigma) as per manufacturer’s instructions. Nuclei were counterstained with DAPI (4′,6-diamidino-2-phenylindole). Cells were visualized and photographed on a fluorescence microscope (Keyence).

### Western blotting

Whole cell extracts were prepared in RIPA buffer [50 mmol/L Tris (pH 8.0), 150 mmol/L NaCl, 0.5% deoxycholate, 0.1% SDS, and 1.0% NP-40] containing protease inhibitor cocktail (Roche). Total protein was electrophoresed by SDS-PAGE and Western blotting was carried out according to standard protocols. The following antibodies were used for Western blotting: CDH1 (Cell Signaling, 3195), Vimentin (Cell Signaling, 5741), SNAI2 (Cell Signaling, 9585), CD44 (Cell Signaling, 3570) and GAPDH (Santa Cruz Biotechnology, sc-32233).

### Luciferase assays

3′UTR regions for CD44 and SLUG containing target sequences complementary to the miR-4287 seed sequence were cloned downstream of the luciferase gene in the pmiR-GLO luciferase vector (Promega). Mutant 3′UTR sequences complementary to miR-4287 were cloned in the same vector. The primers used for clonings were synthesized from Invitrogen and are listed in Supplementary Table 2.

### Statistics

All quantified data represents an average of triplicate samples or as indicated. Data are represented as mean ± S.E.M or as indicated. Two-tailed Student’s *t*-test was used for comparisons between groups. The Wilcoxon Signed Rank test was used to assess the difference between miR-4287 expression in matched tumor/ normal clinical tissues. Correlations between miR-4287 expression and clinicopathological parameters were assessed using Chi squared test. Receiver Operating Characteristic (ROC) curves were generated based on dCt values of miR-4287 in test/control samples. Statistical analyses were performed using MedCalc version 10.3.2. Results were considered statistically significant at *P* ≤ 0.05.

## SUPPLEMENTARY MATERIALS


