# Supplementary material for: MicroRNA-4287 is a novel tumor suppressor microRNA controlling epithelial-to mesenchymal transition in prostate cancer
Source: Oncotarget. 2020 Dec 22;11(51):4681–92. doi: 10.18632/oncotarget.27849 (PMC7771715; doi:10.18632/oncotarget.27849)
Supplement: Supplementary file 1 [file oncotarget-11-4681-s001.pdf]

MicroRNA-4287 is a novel tumor suppressor microRNA controlling epithelial-to mesenchymal transition in prostate cancer

SUPPLEMENTARY MATERIALS

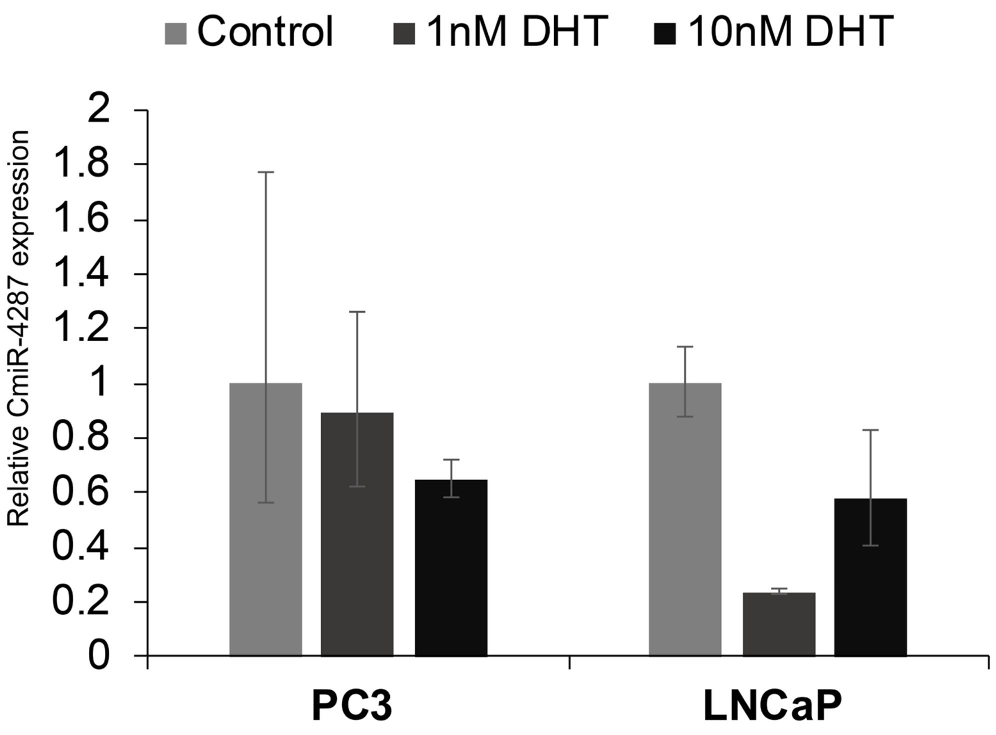

Supplementary Figure 1: miR-4287 expression is repressed by DHT. PC3 and LNCaP cells were treated with 0, 1 and 10 nM DHT followed by real time PCR based expression analyses of miR-4287. RNU48 was used as an endogenous control. Error bars represent SEM.

Supplementary Table 1: Clinicopathological characteristics of prostate cancer clinical samples

Characteristics	Number of patients $N = 68$
Age, Years	
Mean	62.7
Median	62.5
Range	49–79
Race	
Black	31 (46)
White	33 (49)
Other (Asian, American Indian, Native Hawaiian)	1 (1.4)
Unknown	3 (4.4)
T-stage	
pT2 and below	39 (57.3)
pT3	19 (28)
pT4	5 (7.3)
Unknown	5 (7.3)
Gleason Score	
4–6	16 (23.5)
7	33 (48.5)
8–10	19 (28)
PSA	
Median	6.1
< or = 6.6	18 (26.4)
> 6.6	21 (30.8)
Unknown	29 (42.6)
Biochemical recurrence (PSA Failure)	12 (17.6)
N-stage	
N0	16 (23.5)
N1	2 (2.9)
NX	6 (8.8)
Unknown	44 (64.7)
M-stage	3 (4.4)
M0	1 (1.4)
MX	20 (29.4)
Unknown	44 (64.7)
Pathological diagnosis Adenocarcinoma	68 (100)

Supplementary Table 2: List of primers

***CD44*-miR-4287- sense**	5′ AAACTA GCGGCCGC TAGT TTTCACGATAGAAATAAGGGAGG T 3′
***CD44*-miR-4287-antisense**	5′ CTAGA CCTCCCTTATTTCTATCGTGAAA ACTAGCGGCCGC TAGTTT 3′
**Mut *CD44*-miR-4287-2 sense**	5′ AAACTA GCGGCCGC TAGT TTTCACGATAGAAATAACCGGCG T 3′
**Mut *CD44*-miR-4287-2 antisense**	5′ CTAGA CGCCGGTTATTTCTATCGTGAA ACTAGCGGCCGC TAGTTT 3′
***SLUG*-miR-4287- sense**	5′ AAACTA GCGGCCGC TAGT AAAGTATATTTTTAAAACCGGCG T 3′
**Mut *SLUG*-miR-4287- sense**	5′ AAACTA GCGGCCGC TAGT AAAGTATATTTTTAAAAGGGAGG T 3′
***SLUG*-miR-4287-antisense**	5′ CTAGACGCCGGTTTTAAAAATATACTTTACTAGCGGCCGC TAGTTT 3′
**Mut *SLUG*-miR-4287-2 antisense**	5′ CTAGA CCTCCCTTTTAAAAATATACTTT ACTAGCGGCCGC TAGTTT 3′
